# Fabrication of Tough Double-Network Hydrogels from Highly Cross-Linked Brittle Neutral Networks Using Alkaline Hydrolysis

**DOI:** 10.3390/gels10010029

**Published:** 2023-12-28

**Authors:** S. Shams Es-haghi, R. A. Weiss

**Affiliations:** 1 Advanced Structures and Composites Center, The University of Maine, 35 Flagstaff Road, Orono, ME 04469-5793, USA; 2 Department of Chemical and Biomedical Engineering, The University of Maine, 5737 Jenness Hall, Orono, ME 04469-5737, USA; 3 Department of Mechanical Engineering, The University of Maine, 75 Long Road, Orono, ME 04469-5744, USA; 4 Department of Chemical and Biomolecular Engineering, University of Connecticut, 25 King Hill Rd Unit 3136, Storrs, CT 06268-1702, USA

**Keywords:** double-network hydrogels, finite tensile deformation, alkaline hydrolysis, craze-like patterns

## Abstract

This paper describes a simple method to synthesize tough hydrogels from a highly cross-linked neutral network. It was found that applying alkaline hydrolysis to a highly cross-linked hydrogel synthesized from acrylamide (AAm) can increase its swelling ratio dramatically. Double-network (DN) hydrogels synthesized from polymerization of loosely cross-linked AAm networks inside a highly cross-linked AAm gel were not tough. However, repeating the same recipes with a second polymerization step to synthesize a DN hydrogel from a hydrolyzed highly cross-linked AAm gel resulted in tough hydrogels. Those gels exhibited finite tensile behavior similar to that of conventional DN hydrogels. Moreover, craze-like patterns were observed during tensile loading of a DN hydrogel synthesized from a hydrolyzed highly cross-linked first network and a loosely cross-linked second network. The patterns remained in the gel even after strain hardening at high stretch ratios. The craze-like pattern formation was suppressed by increasing the concentration of cross-linking monomer in the second polymerization step. Crack propagation in DN hydrogels synthesized using hydrolysis was also studied by applying a tensile load on notched specimens.

## 1. Introduction

Hydrogels are made from hydrophilic polymer networks that can be highly swollen with water or aqueous solutions [1]. Polymer networks in hydrogels can be made either by chemical or physical cross-links or both [2]. Due to their attractive properties and compatibility with biological tissues, hydrogels can be used in vast areas of applications, like tissue engineering, drug delivery, wound dressing, soft robotics, contact lenses, and flexible bioelectronics [3,4,5,6,7,8,9,10,11,12,13,14,15]. Depending on the application area, hydrogels should have specific physical and mechanical characteristics. In applications where hydrogels are exposed to external loads, they should be mechanically strong and tough. Among various methods to make tough hydrogels, the synthesis of tough chemically cross-linked hydrogels via polymerization of a water-soluble monomer within a highly cross-linked polyelectrolyte gel proposed by Gong et al. [16] opened a new avenue for research in soft, wet materials [17,18,19,20,21,22,23,24,25,26,27,28,29]. This hydrogel architecture, which was initially thought to be a semi-interpenetrating or interpenetrating polymer network (SIPN or IPN) of soft neutral and rigid polyelectrolyte networks, was named a *double-network* (DN) hydrogel. Shams Es-haghi et al. [30] reported infrared spectra evidence for grafting of the polymer chains of the second network to the skeleton of the first rigid network. They also found that the grafting between the two networks is imperative for achieving toughness. In fact, the real architecture of a tough DN hydrogel is either a pseudo-SIPN or pseudo-IPN. 

In the synthesis of conventional, tough DN hydrogels, as developed by Gong et al. [16], the first hydrogel should be a highly cross-linked polyelectrolyte network. In spite of its high cross-link density, a polyelectrolyte gel has a high swelling capacity and therefore can absorb a large amount of the reaction precursor required for synthesizing the second network. In contrast, a DN hydrogel made by polymerization of a neutral first network using the same cross-link density as that used for the polyelectrolyte network is not tough.

Synthesis of tough chemically cross-linked hydrogels without using any ionic network has, however, been reported [31,32]. Argun et al. [31] synthesized tough DN and triple-network (TN) hydrogels from neutral networks using thermal polymerization, and Shams Es-haghi and Weiss [32] developed a simple fabrication process for making tough chemically cross-linked hydrogels from multiple neutral networks. Among these multiple-network hydrogels, the stress–strain behavior of the DN structure is like that of an extensible biological tissue. Being tough and highly extensible, these DN hydrogels exhibit no damage while undergoing finite-strain tensile deformations, even after experiencing freezing and boiling processes [33].

The fabrication of tough chemically cross-linked hydrogels using the DN idea was generalized by Nakajima et al. [34], who showed that by using physically trapped polyelectrolyte chains as molecular stent it is possible to make tough hydrogels from any neutral polymeric network. They used the molecular stent to enhance the swelling capacity of the neutral gel and reported that the molecular stent did not contribute to the mechanical behavior and only increased the swelling ratio of the neutral hydrogel. Shams Es-haghi and Weiss [35] showed that physically trapped polymer chains can contribute to the large-strain tensile mechanics of DN hydrogels. Therefore, molecular stents may actually affect the mechanical response of a DN hydrogel, in contrast to the conclusion reached by Nakajima et al. [34]. This discrepancy may be resolved by comparing the mechanical properties of fresh and aged samples. Diffusion studies on DN hydrogels containing linear polymer chains showed that the linear chains could diffuse out of the DN hydrogels [35,36]. Thus, a reduction of the mechanical properties of the DN hydrogels synthesized using a molecular stent method may be expected. 

The objective of the work described in this paper was to develop an alternative way to synthesize a tough chemically cross-linked hydrogel from a highly cross-linked neutral gel. This was carried out by applying an alkaline hydrolysis process on a weak, highly cross-linked PAAm gel as the first network and then polymerizing a loosely cross-linked neutral network within the first hydrolyzed network. 

## 2. Results and Discussion 

**Figure 1:**
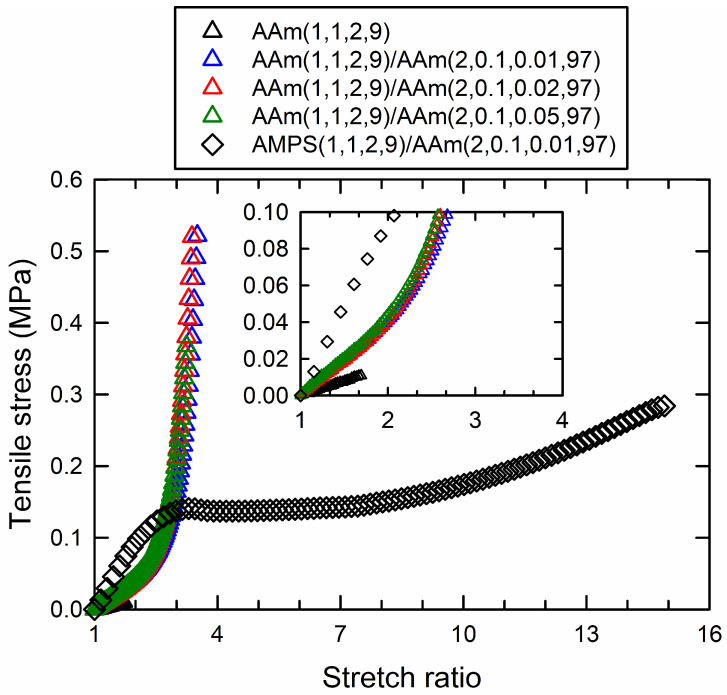
Engineering tensile stress versus stretch ratio for black triangles: single highly cross-linked network (AAm(1,1,2,9)), blue triangles: pseudo-IPN made by polymerization of AAm(2,0.1,0.01,97) inside AAm(1,1,2,9), red triangles: pseudo-IPN made by polymerization of AAm(2,0.1,0.02,97) inside AAm(1,1,2,9), green triangles: pseudo-IPN made by polymerization of AAm(2,0.1,0.05,97) inside AAm(1,1,2,9), and diamonds: pseudo-IPN hydrogel synthesized from AMPS(1,1,2,9) and AAm(2,0.1,0.01,97).

In order to achieve a tough hydrogel using the DN concept, it is necessary that the first network be a highly cross-linked polyelectrolyte network. Polymerization of a loosely cross-linked neutral network inside a highly cross-linked neutral gel does not produce an exceptionally tough hydrogel the way the conventional DN hydrogel synthesis developed by Gong et al. [16] does. Figure 1 compares the tensile mechanical behavior of a highly cross-linked hydrogel made of AAm, three DN hydrogels produced by polymerization of loosely cross-linked neutral networks inside highly cross-linked neutral gels, and a DN hydrogel synthesized from a highly cross-linked polyelectrolyte network, AMPS(1,1,2,9), synthesized with the same recipe used to synthesize the first DN hydrogel from AAm(1,1,2,9).

The highly cross-linked neutral hydrogel AAm(1,1,2,9) was weak. Although the second polymerization step resulted in a drastic change in mechanical behavior of the neutral gel, the DN hydrogels did not exhibit large energy dissipation during tensile deformation. The neutral DN hydrogels were not highly extensible, but their ultimate tensile strength was relatively high. A comparison between mechanical behavior of AAm(1,1,2,9)/AAm(2,0.1,0.01,97) and AMPS(1,1,2,9)/AAm(2,0.1,0.01,97) gels clearly showed the effect of employing a polyelectrolyte network as the first network to achieve a tough hydrogel. Those two DN hydrogels were the same except for the monomer used to synthesize their first networks. The gel synthesized from the polyelectrolyte network underwent plastic deformation and was extended to a high stretch ratio. The imperative role of a highly cross-linked polyelectrolyte network as the skeleton of a DN hydrogel to achieve a high toughness is due to the high swelling ratio of these gels despite their high cross-link density. The high cross-link density of the network resulted in a high modulus at low stretch ratios for the DN hydrogel, and the high swelling ratio absorbed a large amount of second reaction mixture for the second polymerization step. In a tough conventional DN hydrogel, the mass ratio of the second network to the first rigid network is high [16].

**Figure 2:**
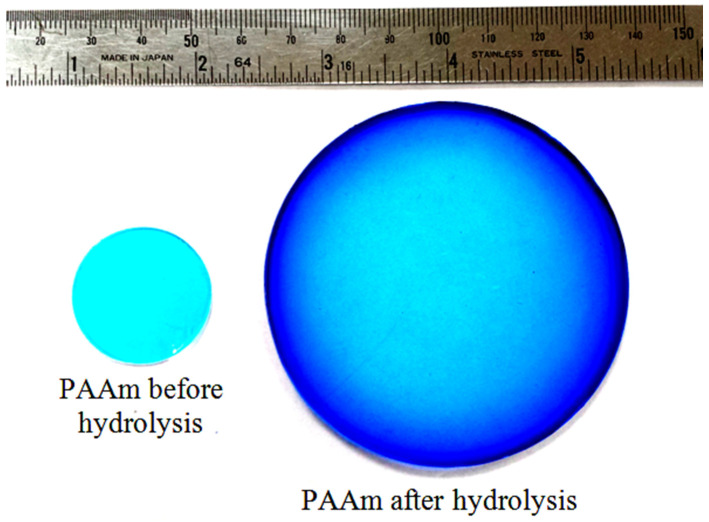
PAAm hydrogel samples colored with methylene blue before and after hydrolysis. Alkaline hydrolysis drastically increases the swelling ratio of a highly cross-linked PAAm gel.

**Figure 3:**
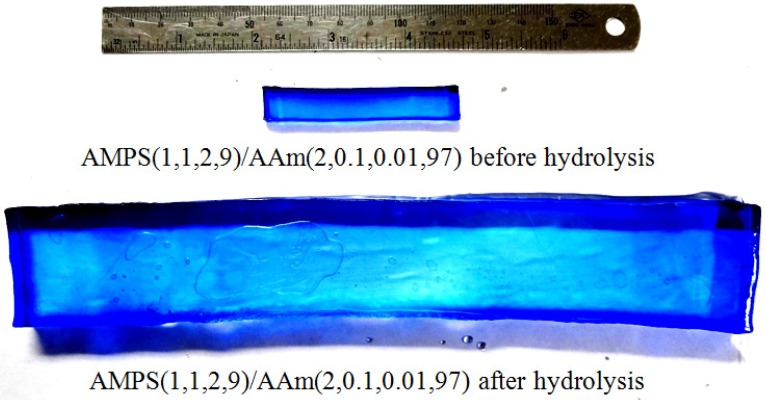
Conventional DN hydrogels colored with methylene blue before and after hydrolysis. Alkaline hydrolysis drastically increases the swelling ratio of a conventional DN hydrogel.

The capacity of a hydrogel to absorb water or an aqueous solution is a quantity that highly depends on the topological microstructure of the gel—more importantly, the cross-link density. However, by changing thermodynamic variables such as temperature and pressure, the swelling ratio of the gel can be manipulated. Among all possibilities to increase the swelling ratio of a cross-linked hydrogel, introduction of ions into the structure of the gel is the most efficient method. Counter ion–ion interactions can significantly increase the osmotic pressure, which accordingly increases the capability of the gel to absorb more water. In the paper by Nakajima et al. [34], introduction of ions was performed by trapping linear polyelectrolyte chains (“molecular stents”) inside the first network, as described in the Introduction section. Herein, we developed a new simple method to increase the swelling ratio of a highly cross-linked neutral gel. This was done by applying an alkaline hydrolysis procedure on the neutral hydrogel. As shown in Section 4.3, by performing the hydrolysis process, a neutral gel became a polyelectrolyte network. Moreover, hydrolysis resulted in a reduction in the average cross-link density by breaking some chemical bonds that eventually increased the swelling ratio of the gel. The possibility of a reduction in the average cross-link density of the neutral PAAm hydrogel due to the hydrolysis process was not considered in a model [37] of the swelling of these gels, and it seems that the authors of Ref. [37] were not aware of our work that was originally reported in Ref. [38]. Figure 2 compares a highly cross-linked hydrogel synthesized from AAm and the same gel after being hydrolyzed using NaOH aqueous solution in their equilibrium swelling states. A drastic increase was observed in the equilibrated volume of the samples. A huge increase in the dimensions of the sample and high water content after the hydrolysis process made the sample more rigid and brittle. The role of high water content, breaking some chemical bonds, and increased rigidity of polymer chains in embrittlement of chemically cross-linked hydrogels can be more easily appreciated by applying the hydrolysis process to a tough conventional DN hydrogel. Figure 3 shows the AMPS(1,1,2,9)/AAm(2,0.1,0.01,97) DN hydrogel before and after hydrolysis. 

**Figure 4:**
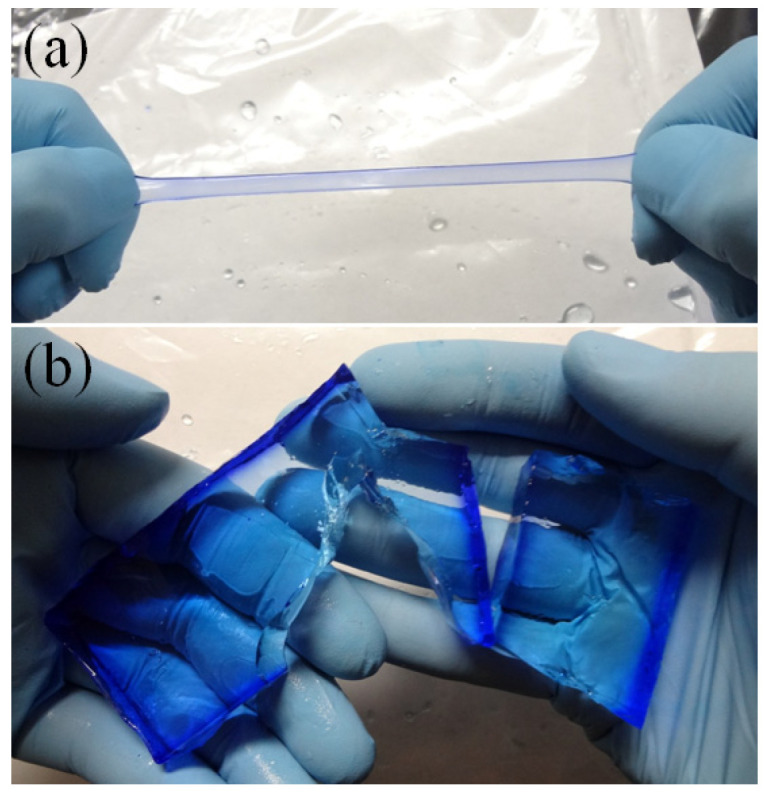
Alkaline hydrolysis makes a tough conventional DN hydrogel brittle. (**a**) AMPS(1,1,2,9)/AAm(2,0.1,0.01,97); (**b**) alkaline-hydrolyzed AMPS(1,1,2,9)/AAm(2,0.1,0.01,97).

After hydrolysis, the swelling ratio of the sample increased markedly. The huge expansion of the sample resulted in the extension of polymer chain strands. Therefore, after hydrolysis, the loosely cross-linked polymer network became rigid. Moreover, the high concentration of water in the hydrolyzed DN hydrogel resulted in a huge reduction in the concentration of the second network. Rigid polymer chains of the second network, breaking some chemical bonds and large water content embrittled the DN hydrogel. Figure 4 shows that, although AMPS(1,1,2,9)/AAm(2,0.1,0.01,97) was tough, after being hydrolyzed the gel became brittle.

**Figure 5:**
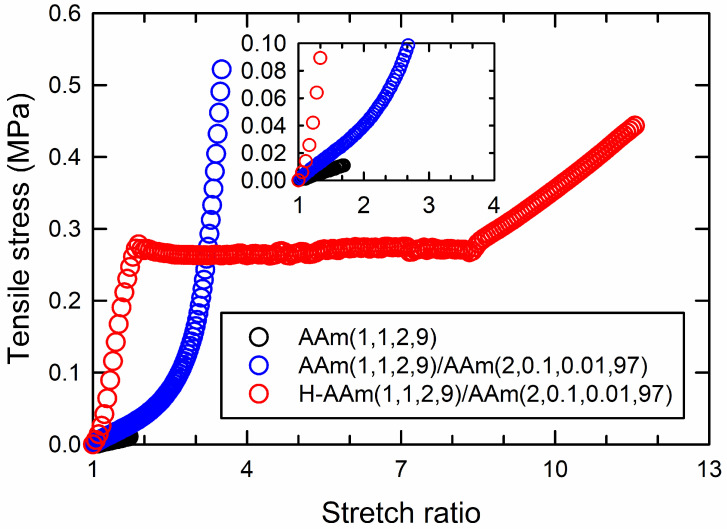
Engineering tensile stress versus stretch ratio for black circles: single highly cross-linked network (AAm(1,1,2,9)), blue circles: pseudo-IPN made by polymerization of AAm(2,0.1,0.01,97) inside AAm(1,1,2,9), and red circles: pseudo-IPN made by polymerization of AAm(2,0.1,0.01,97) inside alkaline-hydrolyzed AAm(1,1,2,9).

Knowing the imperative role of a rigid first network with a high swelling ratio as a skeleton of a tough DN hydrogel, it was expected that the hydrolyzed AAm(1,1,2,9) gel could be used as the first network to create a tough DN hydrogel. Figure 5 compares the mechanical behavior of the AAm(1,1,2,9) single-network gel, AAm(1,1,2,9)/AAm(2,0.1,0.01,97), and a DN hydrogel synthesized from the hydrolyzed AAm(1,1,2,9) gel as the first network and AAm(2,0.1,0.01,97) as the second network. Note that both of the DN hydrogels were the same except for their first networks. Figure 5 shows how using a hydrolyzed highly cross-linked AAm gel resulted in a tough hydrogel. During the hydrolysis process, AAm(1,1,2,9) became more rigid and absorbed more reaction mixture for the second polymerization step compared to the AAm(1,1,2,9) gel. The high modulus of the red data points in Figure 5 is the result of the rigidity of the H-AAm(1,1,2,9) network. The plateau observed during the tensile deformation of H- AAm(1,1,2,9)/AAm(2,0.1,0.01,97) hydrogel is a manifestation of neck propagation during which the engineering tensile stress remains constant.

The high extensibility of the DN hydrogel is due to the high concentration of the second loosely cross-linked gel with long polymer chain strands surrounding the rigid skeleton of the first network that covalently attached to it at some points.

**Figure 6:**
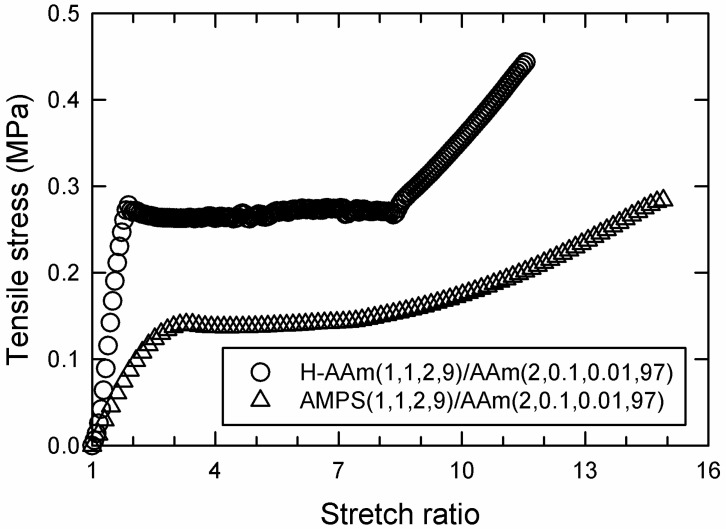
Engineering tensile stress versus stretch ratio for circles: pseudo-IPN made by polymerization of AAm(2,0.1,0.01,97) inside alkaline-hydrolyzed AAm(1,1,2,9) and triangles: pseudo-IPN made by polymerization of AAm(2,0.1,0.01,97) inside AMPS(1,1,2,9).

Figure 6 shows the tensile mechanical behavior of the H-AAm(1,1,2,9)/AAm(2,0.1,0.01,97) and AMPS(1,1,2,9)/AAm(2,0.1,0.01,97) DN hydrogels. The finite tensile behavior of the DN hydrogel synthesized from the hydrolyzed AAm(1,1,2,9) gel was similar to that of the conventional DN hydrogels. Conventional DN hydrogels show damage elastoplasticity with a small plastic strain under tensile loading [39]. A tensile load results in extensive damage to the internal structure of the hydrogel, which manifests in the form of a large hysteresis after unloading. Another important characteristic of convectional DN hydrogels is that they show idealized Mullins effect under finite tensile deformation [40].

**Figure 7:**
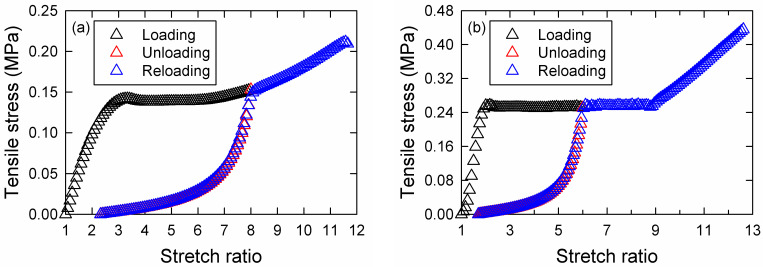
Loading–unloading–reloading tensile tests for (**a**) pseudo-IPN synthesized from AMPS(1,1,2,9) and AAm(2,0.1,0.01,97) and (**b**) pseudo-IPN synthesized from alkaline-hydrolyzed AAm(1,1,2,9) and AAm(2,0.1,0.01,97). Both DN hydrogels exhibit the so-called idealized Mullins effect in finite tensile deformation.

Idealized Mullins effect corresponds to a situation in which a reloading deformation path after unloading from any point of deformation follows the previous unloading path to the stretch ratio of unloading, and then the material resumes its primary loading path [41]. This effect can be characterized by performing loading–unloading–reloading tensile tests. Figure 7 shows the results of loading–unloading–reloading tensile tests on H-AAm(1,1,2,9)/AAm(2,0.1,0.01,97) and AMPS(1,1,2,9)/AAm(2,0.1,0.01,97) DN hydrogels. Both hydrogels showed damaged elastoplasticity with small plastic strain and idealized Mullins effect. 

**Figure 8:**
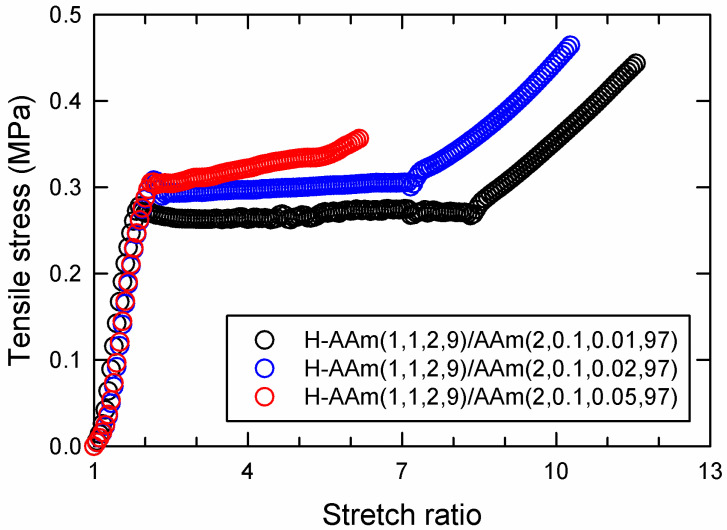
Engineering tensile stress versus stretch ratio for black circles: pseudo-IPN made by polymerization of AAm(2,0.1,0.01,97) inside H-AAm(1,1,2,9), blue circles: pseudo-IPN made by polymerization of AAm(2,0.1,0.02,97) inside H-AAm(1,1,2,9), and red circles: pseudo-IPN made by polymerization of AAm(2,0.1,0.05,97) inside H-AAm(1,1,2,9).

It should be mentioned that the constitutive model developed by Shams Es-haghi and Weiss [42] for the finite tensile deformation of DN hydrogels can also capture the mechanical behavior of the DN hydrogels synthesized by the hydrolysis method [38]. The black circles in Figure 5 show that the AAm(1,1,2,9) single network was a weak hydrogel. H-AAm(1,1,2,9) and AMPS(1,1,2,9) single-network gels were extremely brittle, so they broke in the grips of the tensile machine. Therefore, it was not possible to study the tensile behavior of those gels. However, in a previous study [43], we showed that the elastic contribution of the brittle first network in the tensile mechanics of a DN hydrogel appeared at low stretch ratios in the first regime of deformation before onset of plastic deformation. In effect, by applying a tensile load on the sample, the hard rigid component of the material takes the load. Inspection of the low stretch region in Figure 6 shows a drastic difference between the elastic contribution of the H-AAm(1,1,2,9) and AMPS(1,1,2,9) single networks. Although both gels were prepared using an identical recipe, i.e., the same concentration of components, the hydrolyzed gel was more rigid. Despite the same chemical composition of gels, the difference in preparation might reveal the difference in their rigidity. As explained earlier, the extension of polymer chain strands between cross-links after hydrolysis makes H-AAm(1,1,2,9) rigid. The brittleness due to the extension of polymer chain strands after hydrolysis resulted in an interesting phenomenon. H-AAm(1,1,2,9)/AAm(2,0.1,0.01,97) DN hydrogel exhibited craze-like patterns during tensile deformation. It is important to note that pattern formation was suppressed by increasing the concentration of cross-linking monomer in the second polymerization step to synthesize a DN hydrogel from H-AAm(1,1,2,9). Figure 8 displays the tensile mechanical behavior of pseudo-IPN hydrogels synthesized by polymerization of loosely cross-linked polymer networks with different cross-link density inside the H-AAm(1,1,2,9) hydrogel. All of the samples exhibited necking in tensile loading; however, the intensity of the neck was reduced in the case of H-AAm(1,1,2,9)/AAm(2,0.1,0.05,97). This observation is consistent with our previous finding [39] indicating that finite tensile deformation of DN hydrogels is governed by the cross-link density of the first and second networks. A pseudo-IPN made from a brittle first network may exhibit necking when the second network has a relatively low cross-link density. In a pseudo-IPN hydrogel, necking can be prevented by increasing the cross-link density of the second network. In effect, to synthesize a pseudo-IPN hydrogel that shows strain hardening without necking, the cross-link density of the second network must be adjusted based on the brittleness of the first network [39]. Herein, H-AAm(1,1,2,9) is brittle and AAm(2,0.1,0.05,97) as the second network can reduce the intensity of the neck. Increasing the concentration of cross-linking monomer in the synthesis of the second network also led to a reduction in the stretch ratio at break. This reduction was more pronounced in the case of H-AAm(1,1,2,9)/AAm(2,0.1,0.05,97). 

**Figure 9:**
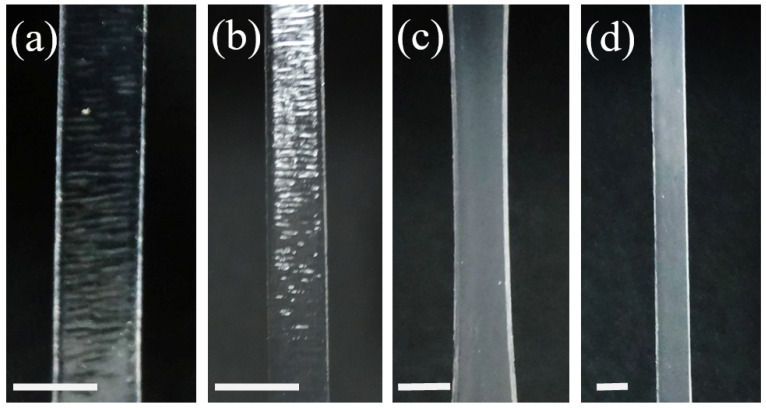
Pseudo-IPN hydrogels polymerized from hydrolyzed AAm(1,1,2,9) network: (**a**) H-AAm(1,1,2,9)/AAm(2,0.1,0.01,97) during tensile loading (stretch ratio: 4); (**b**) H-AAm(1,1,2,9)/AAm(2,0.1,0.01,97) during unloading (stretch ratio: 7); (**c**) H-AAm(1,1,2,9)/AAm(2,0.1,0.02,97) during tensile loading (stretch ratio: 5); (**d**) H-AAm(1,1,2,9)/AAm(2,0.1,0.05,97) during tensile loading (stretch ratio: 5). H-AAm(1,1,2,9)/AAm(2,0.1,0.01,97) exhibits craze-like patterns during tensile loading. The pattern is suppressed by increasing the concentration of cross-linking monomer in the second polymerization step. The scale bars measure 0.5 cm.

Figure 9 shows the appearance of DN hydrogels synthesized from H-AAm(1,1,2,9). The H-AAm(1,1,2,9)/AAm(2,0.1,0.01,97) DN hydrogel exhibited craze-like patterns. Pattern formation was suppressed in the tensile deformation of the H-AAm(1,1,2,9)/AAm(2,0.1,0.02,97) and H-AAm(1,1,2,9)/AAm(2,0.1,0.05,97) DN hydrogels. Both of those gels, with more cross-linked second networks, became completely opaque during deformation. These craze-like patterns are a form of strain localization, which is observed in the tensile loading of polymers like polystyrene and PMMA in their glassy states. In the case of the H-AAm(1,1,2,9)/AAm(2,0.1,0.01,97) DN hydrogel, this phenomenon is attributed to the enhanced brittleness of its first network. The patterns appeared during neck propagation, and they remained in the structure of the hydrogel even at the end of the second regime of deformation, the post-yield regime, before failure. Figure 9b shows the craze-like patterns on a sample during unloading after experiencing strain hardening before failure.

By increasing the concentration of cross-linking monomer in the second polymerization step, the patterns disappeared and the whole sample became opaque. In effect, when the second network was more cross-linked, the increased connectivity between PAAm chain strands reduced the intensity of damage, which was responsible for creating the craze-like patterns. Therefore, homogenized damage occurred in an avalanche process. As mentioned earlier, in a previous study [39], we showed that increasing the cross-link density of the second network can reduce the severity of strain localization in the form of necking in tensile loading. H-AAm(1,1,2,9)/AAm(2,0.1,0.01,97) showed localization phenomena in the form of necking and craze-like patterns. Increasing the cross-link density of the second network could not suppress necking due the high brittleness of the first network; however, the pattern was suppressed.

Shams Es-haghi et al. [43] developed a physical picture to explain the necking phenomenon in DN hydrogels. Based on that model, the finite tensile deformation of a DN hydrogel was characterized based on two regimes of deformation. In the first regime of deformation, the first network that serves as the skeleton for the DN is the load-bearing component. Due to its brittle nature, however, it undergoes a damage process during which a critical point is reached where the second network assumes the load-bearing role. At this point, a neck appears in the sample and a second regime of deformation starts in that the remains of the first network are fractured due to the extension of the PAAm chains. With this in mind, and recalling the earlier explanations in this paper about the similarities of large-strain tensile deformation of conventional DN hydrogels and tough hydrogels synthesized from hydrolyzed highly cross-linked AAm gel, the patterns that appeared in H-AAm(1,1,2,9)/AAm(2,0.1,0.01,97) can be considered as an experimental observation of the fragmentation process of the first network during the second regime of deformation in the tensile mechanics of conventional DN hydrogels. The patterns also provide experimental proof of the estimated internal fracture process presented by Nakajima et al. [44], indicating that the fracture occurs perpendicular to the tensile direction.

**Figure 10:**
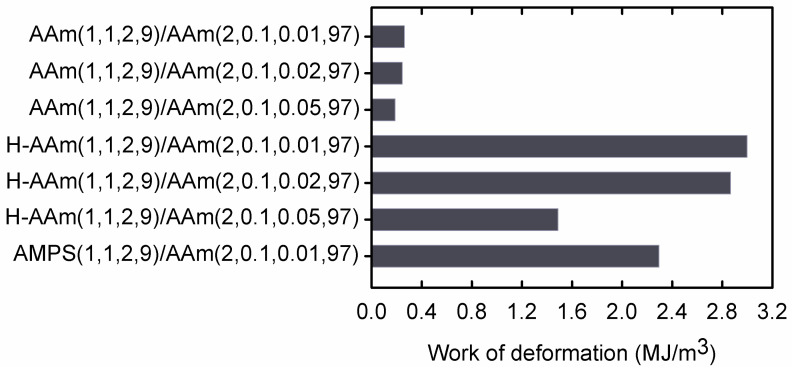
Work of deformation of DN hydrogels synthesized in this study. Work of deformation values were calculated by numerical integration of stress–strain curves.

The work of deformation of all DN hydrogels synthesized in this study was calculated by the integration of stress–strain curves. The work of deformation, which measures the mechanical energy dissipated per unit volume of material during deformation, is a criterion of toughness [45]. Figure 10 summarizes the calculated values. A huge difference between the work of deformation of the DN hydrogels synthesized from AAm(1,1,2,9) and that of the DN hydrogels synthesized from H-AAm(1,1,2,9) clearly shows the effectiveness of the hydrolysis process in achieving a tough hydrogel. 

**Figure 11:**
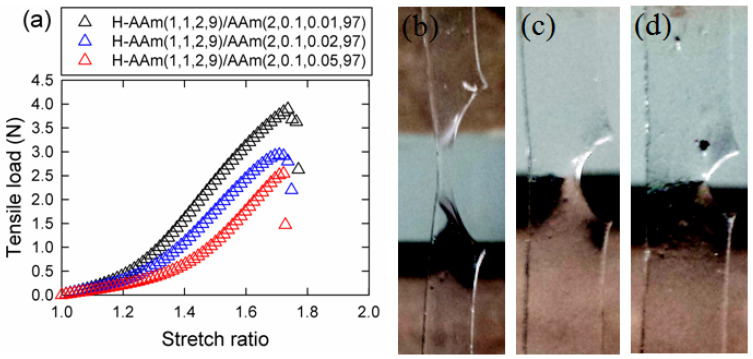
Tensile loading of notched samples: (**a**) tensile load versus stretch ratio; (**b**) H-AAm(1,1,2,9)/AAm(2,0.1,0.01,97) at the onset of failure; (**c**) H-AAm(1,1,2,9)/AAm(2,0.1,0.02,97) at the onset of failure; (**d**) H-AAm(1,1,2,9)/AAm(2,0.1,0.05,97) at the onset of failure.

The crack propagation in DN hydrogels synthesized from hydrolyzed highly cross-linked AAm gel was studied by applying a tensile load on notched specimens. Figure 11 shows the variation in tensile load and crack propagation in samples at the onset of failure. Crack propagation occurred quickly in the samples. In the case of the H-AAm(1,1,2,9)/AAm(2,0.1,0.01,97) DN hydrogel, a neck-like region appeared, but it did not propagate and immediately broke. In samples with a more cross-linked second network, the crack propagation was quick. It is interesting to note that all of the notched DN hydrogels remained transparent during tensile loading. In effect, rapid crack propagation at low stretch ratios prevented the breakage of the rest of the sample, and therefore, the sample remained transparent. The crack propagation observed herein is consistent with the results of our previous work where we studied crack propagation in pseudo-IPN conventional DN hydrogels [39].

## 3. Conclusions

In order to achieve a tough conventional double-network (DN) hydrogel, the first gel must be a highly cross-linked polyelectrolyte network. A DN hydrogel synthesized by polymerization of a loosely cross-linked polymer network inside a highly cross-linked neutral gel cannot dissipate the energy of deformation. We developed a simple method to fabricate tough hydrogels from a highly cross-linked neutral polymeric network. In this method, after applying an alkaline hydrolysis process on a highly cross-linked AAm gel by immersing it in a 1 M aqueous solution of NaOH, the swelling ratio of the gel increased drastically. Polymerization of loosely cross-linked networks inside that hydrolyzed gel produced tough hydrogels. The finite tensile behavior of the DN hydrogel synthesized from a hydrolyzed highly cross-linked gel was similar to that of conventional DN hydrogels. Those DN hydrogels exhibited damage elastoplasticity with small plastic strain, and idealized Mullins effect was observed after performing loading–unloading–reloading tensile tests. Moreover, the crack propagation in DN hydrogels synthesized from a hydrolyzed first network was similar to the crack propagation in conventional DN hydrogels. 

The DN hydrogel synthesized by polymerization of a loosely cross-linked second network, AAm(2,0.1,0.01,97), inside a hydrolyzed highly cross-linked gel exhibited craze-like patterns during neck propagation. The formation of patterns is a manifestation of strain localization and is attributed to the enhanced brittleness of the first network due to the extension of polymer chain strands. The patterns remained in the structure of the hydrogel after unloading the sample at high stretch ratios. The craze-like patterns provide experimental proof of the fragmentation of the first network during neck propagation. By increasing the concentration of cross-linking monomer in the second polymerization step, the patterns were suppressed and the whole sample became opaque. In effect, when the second network was more cross-linked, the increased connectivity between PAAm chain strands reduced the intensity of the damage that was responsible for creating the craze-like patterns and, therefore, homogenized damage occurred in an avalanche process and the whole sample became cloudy.

## 4. Experimental Section

### 4.1. Materials

Materials including 2-acrylamido-2-methylpropane sulfuric acid (AMPS) and acrylamide (AAm) were obtained from Sigma-Aldrich Chemical Co. (Milwaukee, WI, USA) and used as received. A photoinitiator, 2-oxoglutaric acid (OXGA), and sodium hydroxide (NaOH) were purchased from Fluka Chemical Co. and used as received. The cross-linking monomer *N*,*N*′-methylenebis(acrylamide) (MBAA) was obtained from Sigma-Aldrich Chemical Co. (Milwaukee, WI, USA) and was recrystallized from ethanol. 

### 4.2. Synthesis of Highly Cross-Linked PAAm Hydrogel

**Scheme 1:**
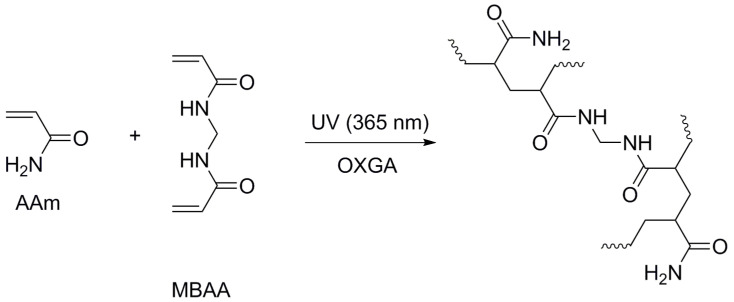
Synthesis of poly(acrylamide) gel.

Highly cross-linked PAAm gels were synthesized by adding MBAA and OXGA to a 1 M solution of AAm in deionized (DI) water. To remove oxygen from the reaction mixture, it was bubbled with dry nitrogen gas for 5–10 min and the reaction solution was then injected into a glass mold made of two parallel glass slides that was then exposed for 10 min to 365 nm ultraviolet (UV) light (15 mW/cm^2^) (Scheme 1). 

### 4.3. Alkaline Hydrolysis of PAAm Hydrogel

**Scheme 2:**
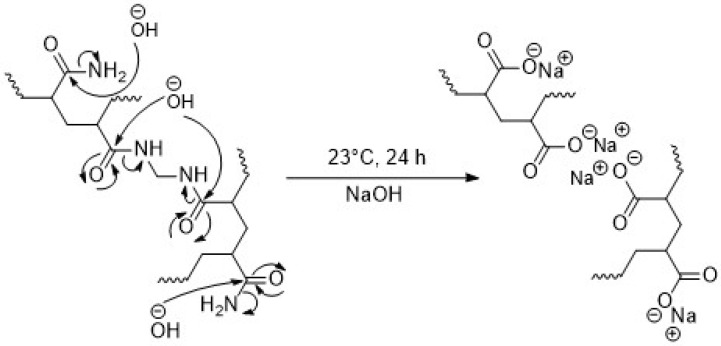
Alkaline hydrolysis of poly(acrylamide) gel.

The PAAm gel was immersed in 1 M aqueous solution of NaOH at 23 °C for 24 h (Scheme 2). Then, the gel was washed at least 5 times with fresh DI water over a period of 3 days and then dried. 

### 4.4. Synthesis of Pseudo-IPNs from Hydrolyzed and Neutral PAAm Gels

The resulting hydrolyzed hydrogel was submerged in a 2 M aqueous solution of AAm with MBAA containing the photoinitiator (0.1 mol % with respect to the AAm) that had already been bubbled with nitrogen. After reaching the equilibrium swelling condition, the AAm-swollen gel was placed between two parallel glass slides and exposed for 9 h to 365 nm UV light (3 mW/cm^2^) (Scheme 1). Pseudo-IPNs were also synthesized from the PAAm gels with the same procedure without the hydrolysis process. 

### 4.5. Synthesis of a Pseudo-IPN from PAMPS Gel

**Scheme 3:**
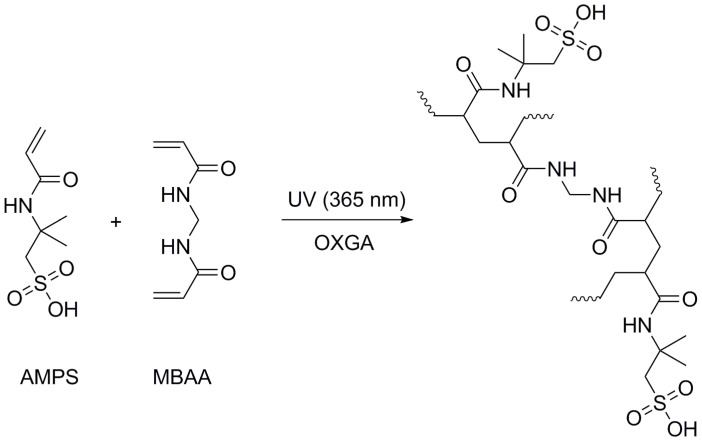
Synthesis of the PAMPS network.

A pseudo-IPN hydrogel was synthesized from polyAMPS gel as the first network with the same recipe used for making DN hydrogel from AAm. The first network was prepared by adding MBAA and OXGA to a 1 M aqueous solution of AMPS. To remove oxygen from the reaction mixture, it was bubbled with dry nitrogen gas for 5–10 min, and the reaction solution was then injected into a glass mold made of two parallel glass slides that was then exposed for 10 min to 365 nm ultraviolet (UV) light (15 mW/cm^2^) (Scheme 3). The resulting AMPS gel was then submerged in a 2 M aqueous solution of AAm, with MBAA (0.01 mol % with respect to the AAm) containing the photoinitiator that had already been deoxygenated with nitrogen. When the hydrogel reached the equilibrium swelling condition, the AAm-swollen AMPS gel was placed between two parallel glass slides and exposed for 9 h to 365 nm UV light (3 Mw/cm^2^) (Scheme 1). One DN hydrogel was hydrolyzed with the same procedure described above for AAm gels.

The resulting DN hydrogel samples were immersed in DI water, which was replaced several times with fresh DI water to remove any unreacted monomer. The sample notation contains information about the recipes used for making different networks of hydrogels. Each network is shown with the name of the monomer utilized to synthesize the polymeric network followed by four numbers in parenthesis that show (a) the monomer molar concentration in water, (b) the mol% of OXGA with respect to the monomer, (c) the mol % of MBAA with respect to the monomer, and (d) the UV dose used for the reaction (i.e., exposure time x intensity). In the case of hydrolyzed first networks, the H- prefix was used before the name of the first network. Thus, AAm(1,1,2,9) corresponds to the polymerization of a 1 M AAm solution using 1 mol % OXGA, 2 mol % MBAA, and a UV dose of 9 J/cm^2^. H-AAm(1,1,2,9) denotes the hydrolyzed AAm(1,1,2,9). All DN hydrogels synthesized and used in this study are summarized in Table 1.

### 4.6. Tensile Experiments

An Instron 5567 universal testing machine equipped with a 100 N load cell was used to perform uniaxial tensile experiments. All of the experiments were conducted using a constant crosshead speed of 50 mm/min at room temperature. Rectangular samples (40 mm long, 10 mm wide, and 2–3 mm thick) and a gauge length of 30 mm were used to perform the experiments. Sandpaper was used in the grips of the tensile machine to prevent slippage of the hydrogel samples. The results of the tensile experiments are reported as engineering tensile stress versus stretch ratio, where the engineering tensile stress is the ratio of the tensile force to the original cross-sectional area of the sample and the stretch ratio is the ratio of the instantaneous length to the original length of the sample.

Crack propagation in pseudo-IPN hydrogels synthesized using the hydrolysis process was studied with single-edge notch fracture experiments. A 1 mm notch was made in the center of one of the sample edges, and the sample was then deformed by applying a tensile deformation with an extension speed of 50 mm/min.

## Figures and Tables

**Table 1:** DN hydrogels synthesized in this study.

DN Hydrogel Notation	Formulation of First Network (pphm) ^a^			Formulation of Second Network (pphm) ^a^			First Network/ Second Network
	OXGA	MBAA	UV Dose (J/cm^2^)	OXGA	MBAA	UV Dose (J/cm^2^)	
AAm(1,1,2,9)/ AAm(2,0.1,0.01,97)	1	2	9	0.1	0.01	97	Neutral/Neutral
AAm(1,1,2,9)/ AAm(2,0.1,0.02,97)	1	2	9	0.1	0.02	97	Neutral/Neutral
AAm(1,1,2,9)/ AAm(2,0.1,0.05,97)	1	2	9	0.1	0.05	97	Neutral/Neutral
H-AAm(1,1,2,9)/ AAm(2,0.1,0.01,97)	1	2	9	0.1	0.01	97	Hydrolyzed/Neutral
H-AAm(1,1,2,9)/ AAm(2,0.1,0.02,97)	1	2	9	0.1	0.02	97	Hydrolyzed/Neutral
H-AAm(1,1,2,9)/ AAm(2,0.1,0.05,97)	1	2	9	0.1	0.05	97	Hydrolyzed/Neutral
AMPS(1,1,2,9)/ AAm(2,0.1,0.01,97)	1	2	9	0.1	0.01	97	Polyelectrolyte/Neutral

^a^ Parts per hundred parts monomer.

## Data Availability

The data that support the findings of this study are available from the corresponding author upon reasonable request.
